# Dr. Lankester on Food

**Published:** 1859-08

**Authors:** 


### DR. LANKESTER ON FOOD.

It cannot be denied that the human body is influenced at least as powerfully by the food we take, as by the medicine which we more rarely and unwillingly swallow. A course of lectures on food, therefore, seems a most useful and important preliminary to a course on materia medica. This subject is, however, usually slurred over, or very imperfectly handled, by lecturers on materia medica. The natural result is, that the study of food is considerably neglected by medical men, while by the general laity it is almost wholly ignored. Dr. Lankester has long been a prominent and eloquent exponent of the chemical, economic and dietetic relations of food; and when the elevation of Dr. Lyon Playfair to the Chemical

Chair in the University of Edinburgh rendered vacant the office of Superintendent of the Animal Product and Food Collections at the South Kensington Museum, Dr. Lankester was named as an able successor in that office. With characteristic energy, he set himself to forward the active development of these most interesting collections, and has resolved to make them as fully available as may be for general instruction. By the permission of the Committee of the Council of Education, he has initiated a course of six lectures on Food, to be delivered in the Lecture Theatre, at South Kensington, on Monday evenings, the 2d, 9th, 16th, 23d and 30th of May, and 6th of June, at 8 o'clock, at merely a nominal fee. The object of the course will be to explain the nature and sources of human food; and, with this view, the chemical properties are demonstrated, and the natural history of plants yielding food described and illustrated. The first lecture has been delivered: the subject was Water. It is difficult for those who are not personally cognizant—as, however, so many are—of the admirable endowments which the lecturer possesses, to imagine the eloquence, the humor, the philosophic generalizations, and the occasional touches of gravity, which rendered this lecture as attractive to the audience as it was replete with sound views and valuable teachings. The subsequent lectures, including the doctrines of nutrition and animal heat, with an examination of all the botanical and biologic relations of starch and sugar, flesh-forming foods, alcoholic compounds, tea, coffee, and chocolate, will deal with subjects which might furnish ample material for a much fuller course. We commend these lectures, and the very interesting collections at South Kensington, alike to the notice of students and practitioners.—*London Lancet.*

